# Divergent Mechanisms of Antidepressant Efficacy: A Unified Computational Comparison of Synaptogenesis, Stabilization, and Tonic Inhibition in a Model of Depression

**DOI:** 10.7759/cureus.105040

**Published:** 2026-03-11

**Authors:** Ngo Cheung

**Affiliations:** 1 Psychiatry, Cheung Ngo Medical Limited, Hong Kong, HKG

**Keywords:** computational psychiatry, depression, ketamine, neurosteroid, selective serotonin reuptake inhibitors (ssris)

## Abstract

Background: Major depressive disorder (MDD) is increasingly viewed as a disorder of impaired neural plasticity, yet the mechanisms underlying diverse antidepressant classes - glutamatergic (e.g., ketamine), monoaminergic (e.g., selective serotonin reuptake inhibitors (SSRIs)), and GABAergic (e.g., neurosteroids) - remain incompletely integrated. The objective of this study was to extend a pruning-plasticity model of depression and directly compare, from an identical severely pruned baseline state, the efficacy, stress resilience, durability, and relapse vulnerability of three mechanistically distinct interventions: ketamine-like targeted synaptogenesis, SSRI-like gradual refinement of existing connectivity, and neurosteroid-like tonic inhibition. Computational models offer a controlled means to compare these pathways, but prior work has typically examined single mechanisms.

Methods: We extended a pruning-plasticity model of depression by applying 95% magnitude-based synaptic elimination to overparameterized feed-forward networks trained on a four-class Gaussian classification task. From identical pruned states, three interventions were tested: ketamine-like gradient-guided regrowth (50% reinstatement) with consolidation; SSRI-like prolonged low-learning-rate training with gradual internal noise reduction; and neurosteroid-like global tonic inhibition (30% damping plus tanh activations) with brief consolidation. Outcomes included baseline accuracy, resilience to graded internal activation noise (up to σ = 2.5) plus input perturbation, and relapse vulnerability after an additional 40% pruning.

Results: All treatments restored near-ceiling performance on unchallenged inputs. Ketamine-like synaptogenesis uniquely reduced sparsity (to ~47%) and conferred superior stress resilience (extreme noise accuracy 84.5%) with near-zero relapse drop (−0.2%). SSRI-like refinement improved combined stress accuracy to 83.5% but showed limited extreme noise tolerance (44.0%) and substantial relapse vulnerability (10.8% drop). Neurosteroid-like inhibition achieved rapid combined stress recovery (97.5%) while active, but was state-dependent (decline upon removal) with poor extreme noise buffering (42.5%) and moderate relapse drop (4.1%).

Conclusions: These simulations demonstrate that antidepressants operate through mechanistically distinct routes-structural rebuilding (ketamine), gradual optimization of existing connectivity (SSRIs), or reversible dynamic stabilization (neurosteroids)-yielding trade-offs in onset speed, durability, and stress resilience. The findings support a multifaceted plasticity framework for depression and provide computational rationale for mechanism-based treatment selection and combination strategies.

## Introduction

Major depressive disorder (MDD) is a top driver of disability worldwide, touching hundreds of millions of people and creating large social and economic burdens [1]. Drug treatment has helped many, yet results are still disappointing: only about one-third of patients feel well after an initial medicine, and roughly another third remain ill even after trying several options [2]. The most common medicines - selective serotonin reuptake inhibitors (SSRIs) - often take weeks to work, leaving patients unprotected during that wait and, for many, never bringing full relief [3].

This slow and uneven response has pushed scientists to look for faster and more reliable approaches. One such option is ketamine, a drug that blocks N-methyl-D-aspartate (NMDA)-type glutamate receptors. In many studies, a single low-dose infusion eases mood and even suicidal thoughts within hours, an effect tied to a quick burst of new synapses through brain-derived neurotrophic factor (BDNF) and mammalian target of rapamycin (mTOR) signaling [4,5]. Neuroactive steroids such as brexanolone and zuranolone, which raise tonic gamma-aminobutyric acid type A (GABA_A) inhibition, can also lift depression quickly, especially after childbirth [6]. Neurosteroids act as positive allosteric modulators of extrasynaptic GABA_A receptors (particularly δ-subunit-containing), enhancing tonic inhibition and rapidly restoring excitatory-inhibitory balance in corticolimbic circuits without requiring synaptic structural change [7].

Findings like these challenge the classic "low-serotonin" story and instead point to problems in brain plasticity: long-term stress thins dendrites and prunes synapses in key regions like the prefrontal cortex and hippocampus, reducing the brain's flexibility when new stress appears [8,9].

Computational psychiatry integrates multi-scale data and mechanistic models to generate testable hypotheses that are difficult to examine directly in humans [10,11]. Previous plasticity-based models of depression have shown that excessive synaptic pruning creates networks that appear intact at rest but collapse under noise or further challenge [12,8,13]. The current study extends this framework by directly comparing three mechanistically distinct antidepressant interventions from an identical pruned baseline.

Computer models help explore how such circuit changes might lead to illness. One model borrows the idea of excess teenage pruning, first proposed for schizophrenia, and applies it to depression. In this view, trimming too many synapses leaves networks that look fine at rest but fall apart when noise or stress is added [12]. Letting the network regrow the most useful connections restores function without returning to full density, echoing the way ketamine sparks limited but targeted synaptogenesis. What is still unclear is why glutamatergic, monoaminergic, and GABAergic treatments differ in how fast they act, how long they last, and which patients they suit best.

To tackle that question, we extend the pruning-plasticity model and compare three stand-ins: (1) a ketamine-like surge of targeted synapse growth, (2) an SSRI-like slow strengthening of the remaining links, and (3) a neurosteroid-like boost in baseline inhibition. Starting with the same over-pruned network, we watch how each strategy aids basic recovery, guards against later stress, and affects the chance of relapse. The results should clarify each drug class's trade-offs and guide more personal treatment choices as the field turns toward rapid, plasticity-focused therapies.

## Materials and methods

Network architecture and task

**Figure 1:**
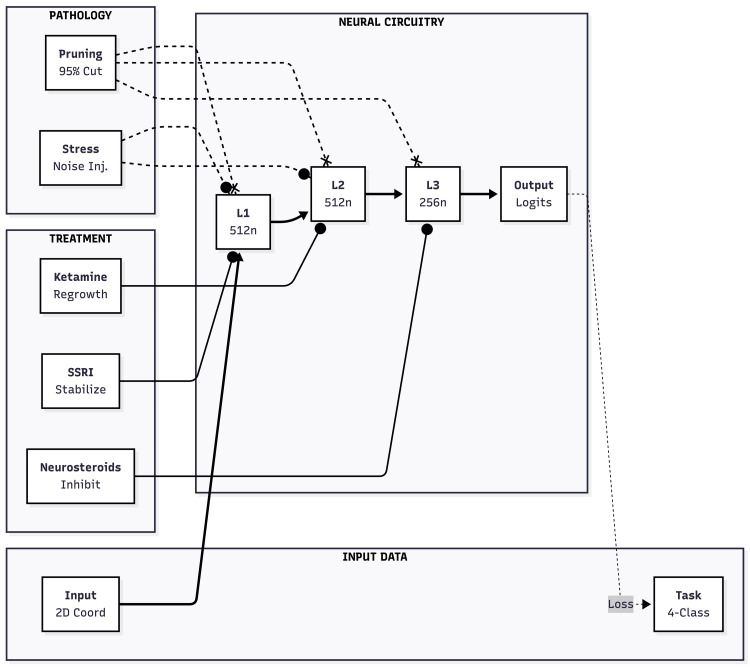
Schematic representation of the neural circuitry model, pathological mechanisms, and therapeutic interventions The central pipeline (Neural Circuitry) consists of a three-layer feed-forward network processing 2D coordinate inputs. Pathology modules (dashed lines) introduce synaptic pruning (95% sparsity) and stress-induced noise into the hidden layers. Treatment modalities (solid lines) represent distinct pharmacological mechanisms: Ketamine induces gradient-guided regrowth in Layer 2, SSRIs stabilize weights in Layer 1, and GABAergic neurosteroids provide tonic inhibition in Layer 3. Image credits: Ngo Cheung

To study circuit fragility and recovery, we built a fully connected feed-forward network that stands in for the mood-relevant cortex (Figure 1). The model accepted two continuous inputs, passed activity through three hidden layers of 512, 512, and 256 ReLU units, and produced four softmax outputs. Altogether, it held about 397,000 trainable weights. Two features captured neuromodulatory influence: (1) additive Gaussian noise could be injected after each hidden layer, and (2) a global post-activation scaling factor could damp overall firing, mimicking tonic inhibition.

The classification problem involved four Gaussian clusters centred at (-3, -3), (3, 3), (−3, 3), and (3, -3) with a standard deviation of 0.8. We created 12,000 training points, 4,000 noisy test points, and 2,000 clean test points. This simple task allowed a clear read-out of how pruning and noise hurt accuracy and how different "treatments" repaired it.

Study design

This study employed an experimental computational modeling design. All simulations were performed between 1 January and 31 January 2026 using PyTorch (Meta AI, USA) on Google Colab (Google Research, USA).

Baseline training and pruning

Networks started from random weights and were trained for 20 epochs with Adam (learning rate = 0.001) while internal noise was set to zero. After the dense model reached stable performance, we removed the smallest 95% of weights in each layer. The resulting 95%-sparse network still handled clean inputs but failed under noise, paralleling the hidden liability proposed for major depressive disorder.

The network was trained using categorical cross-entropy loss L = -sum(y_i * log(ŷ_i)) for i=1 to C, with a batch size of 128. Top-1 classification accuracy was the sole evaluation metric, defined as Accuracy = (number of correct predictions / total samples) × 100. No additional regularization techniques (e.g., dropout, weight decay, or L2 penalty) were applied beyond the Adam optimizer defaults. The 95% magnitude-based pruning threshold was chosen because prior simulations [12] established it as the critical fragility threshold: networks retain near-normal clean-input performance yet collapse under modest internal noise, faithfully reproducing the "intact at rest, fragile under stress" phenotype proposed for MDD.

Treatment protocols

**Figure 2:**
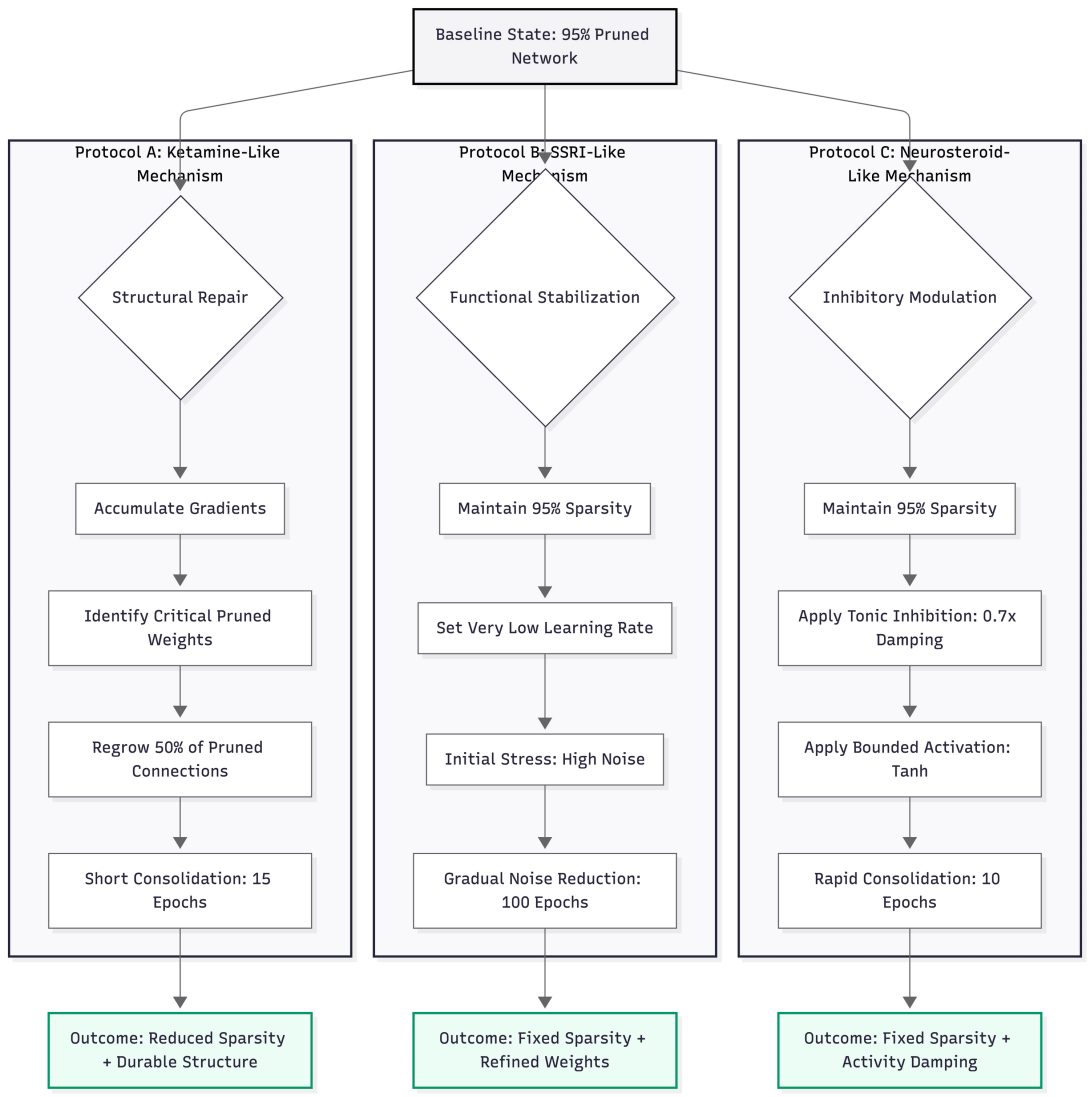
Comparative computational protocols for antidepressant mechanisms The diagram illustrates the procedural logic for the three simulated treatments starting from an identical baseline (95% pruned/depressed state). Protocol A (Ketamine-like): focuses on structural plasticity. The model accumulates gradients to identify "ghost" connections that would be beneficial if restored, then explicitly regrows 50% of them, followed by a brief consolidation period. Protocol B (SSRI-like): focuses on gradual functional adaptation. The network structure remains fixed (sparse). The model undergoes a long training period (100 epochs) with a very low learning rate while internal noise (stress) is linearly tapered from 0.5 to 0.0. Protocol C (Neurosteroid-like): focuses on rapid inhibitory modulation. Structure remains fixed. The network's forward pass is modified to include multiplicative damping (0.7x) and bounded activation functions (Tanh), simulating enhanced tonic GABAergic inhibition. Image credits: Ngo Cheung

Three recovery procedures were applied to identical copies of the pruned model (Figure 2).

Ketamine-like recovery re-added 50% of the lost weight. Candidates for regrowth were chosen by their gradient size, calculated over 30 mini-batches without noise. New weights were drawn from a normal distribution with mean 0 and SD 0.03. A binary mask locked sparsity in place while the network was fine-tuned for 15 epochs with Adam (learning rate = 0.0005).

SSRI-like recovery left the sparse structure untouched and relied on slow parameter drift. The model was trained for 100 epochs at a learning rate of 1 × 10⁻⁵. Internal activation noise began at σ = 0.5 and declined linearly to zero, representing gradual neurotransmitter stabilisation.

Neurosteroid-like recovery also kept the sparse mask but altered activation dynamics. A global damping factor of 0.7 was applied after each hidden layer, and ReLU activations were replaced with tanh to cap firing rates. Ten epochs of tuning at 0.0005 allowed the model to settle. Performance was later checked with the damping switch on (drug present) and off (drug withdrawn).

Parameter choices were selected to achieve comparable acute recovery of clean accuracy while faithfully reflecting known pharmacological properties. The 50% reinstatement in the ketamine-like protocol approximates the magnitude of rapid synaptogenesis and spine density increase (~20-50%) reported in preclinical ketamine studies [4,14]. The prolonged 100-epoch training at 1 × 10⁻⁵ learning rate with linear noise taper in the SSRI-like protocol models the slow downstream neuroadaptive processes (BDNF signalling, receptor desensitisation) characteristic of monoaminergic antidepressants [15]. The global damping factor of 0.7 (≈30% reduction in firing) together with the switch to tanh activations in the neurosteroid-like protocol directly simulates the enhancement of tonic GABAergic conductance produced by neurosteroids [6,7].

Iso-dose matching analysis

To control for unequal network alteration across protocols, an iso-dose comparison was performed. For each treatment, systematic parameter sweeps were conducted (e.g., regrowth percentage, learning rate, damping factor). The L1 norm of weight differences (normalised per parameter) served as a cross-mechanism "dose" proxy. Configurations with overlapping L1 values (range ≈0.000855-0.007719) were selected for direct comparison of acute recovery, stress resilience, and relapse vulnerability.

Evaluation and stress tests

Accuracy was recorded on three test sets: clean, original noisy, and combined stress (input noise σ = 1.0 plus internal noise σ = 0.5). We also swept internal noise from σ = 0.3 to 2.5 to chart resilience. To mimic relapse, an extra 40% of the remaining weights were pruned after treatment; the combined-stress test was then repeated.

Reproducibility

All runs used seed 42 for weight initialisation, data sampling, and noise generation. Experiments were implemented in Python 3.12 using PyTorch 2.2 on Google Colab CPU runtime. All modeling, analysis, and scientific content are the sole work of the author. Code is available from the authors at https://github.com/cheungngo/Divergent-Mechanisms-of-Antidepressant-Efficacy.

## Results

Baseline performance of the pruned network

Removing 95% of the weights immediately exposed the model's latent weakness. Accuracy on noise-free inputs slipped to 50.8%, and the usual test set that already contained input jitter registered only 43.9%. When the combined challenge of input noise (σ = 1.0) and internal activation noise (σ = 0.5) was applied, accuracy fell further to 31.8%. A sweep of pure internal noise showed the steepest losses: under extreme noise (σ = 2.5), the network managed just 28.3%. These values set the benchmark for all later comparisons.

Effects of ketamine-like treatment

Restoring half of the pruned synapses with gradient targeting cut sparsity to 47.5%. After 15 fine-tuning epochs, the network again scored 100% on both clean and standard inputs. Robustness also rebounded; combined-stress accuracy reached 96.9%, and even under extreme internal noise, the system held at 84.5%. When a second pruning wave (40% of the remaining weights) was imposed to mimic relapse, combined-stress accuracy dipped by only 0.2%, revealing virtually complete protection.

Effects of SSRI-like treatment

Keeping the 95% sparsity unchanged but training slowly with fading internal noise restored 100% clean accuracy and 99.5% on the standard test set. Stress tolerance improved but did not match the ketamine analogue: combined-stress accuracy climbed to 83.5%, and performance under extreme internal noise plateaued at 44.0%. After the relapse-style pruning, combined-stress accuracy dropped a further 10.8%, indicating a sizeable vulnerability once additional damage occurred.

Effects of neurosteroid-like treatment

Introducing a 0.7 global damping factor and tanh activations, while still 95% sparse, yielded 100% accuracy on clean and standard inputs when the modulation was active. Combined-stress accuracy peaked at 97.5%, close to the ketamine result, yet extreme internal noise remained difficult (42.5%). Turning the damping off-simulating drug withdrawal-lowered combined-stress accuracy to 90.5% but paradoxically raised extreme-noise accuracy to 58.6%, showing that state dependence shaped resilience. A relapse challenge under active modulation produced a modest 4.1% decline, midway between the ketamine and SSRI patterns.

Comparative summary

All three interventions restored perfect or near-perfect behaviour on unperturbed data, but their stress profiles diverged (Tables 1, 2). Structural regrowth delivered by the ketamine-like procedure offered the strongest buffer against both high noise and a second pruning insult. The neurosteroid model nearly matched the ketamine condition on the main stress test but relied on the continued presence of modulation and showed weaker tolerance of extreme internal noise. The SSRI-like strategy achieved respectable gains yet remained the most fragile when additional pruning simulated relapse.

**Table 1:** Post-treatment performance and relapse vulnerability across conditions. Note: Values represent classification accuracy (%). "Standard" refers to standard noisy test data. "Combined Stress" includes input noise (σ = 1.0) plus internal activation noise (σ = 0.5). Relapse Drop reflects the percentage point decrease in combined stress accuracy following an additional 40% pruning of remaining weights. Dashes (—) indicate metrics not applicable to the specific experimental condition. Standard noisy test data refers to the held-out test set containing input perturbation (σ = 0.8 cluster noise). For the neurosteroid-like (off) condition, clean, standard, and relapse-drop values are not reported because performance metrics in these conditions are only meaningful while the inhibitory modulation is active.

Condition	Sparsity (%)	Clean (%)	Standard (%)	Combined stress (%)	Extreme stress (σ = 2.5) (%)	Relapse drop (%)
Untreated (pruned)	95.0	50.8	43.9	31.8	28.3	—
Ketamine-like	47.5	100.0	100.0	96.9	84.5	−0.2
SSRI-like	95.0	100.0	99.5	83.5	44.0	10.8
Neurosteroid-like (on)	95.0	100.0	100.0	97.5	42.5	4.1
Neurosteroid-like (off)	95.0	—	—	90.5	58.6	—

**Table 2:** Accuracy (%) under increasing internal activation noise. Note: Data reflect performance resilience against graded internal noise levels without additional input perturbation.

Condition	No noise	Moderate (σ = 0.5)	High (σ = 1.0)	Severe (σ = 1.5)	Extreme (σ = 2.5)
Untreated (pruned)	43.9	31.6	29.9	28.0	28.3
Ketamine-like	100.0	99.8	99.0	96.1	84.5
SSRI-like	99.5	87.2	70.3	58.0	44.0
Neurosteroid-like (on)	100.0	99.9	89.6	69.3	42.5

## Discussion

Interpretation of results

The simulations paint three clearly different recovery pictures that help explain why patients respond so unevenly to modern antidepressants. All modelled treatments returned the network to flawless performance when no stressor was present, mirroring the clinical reality that most drugs eventually lift core symptoms in people who respond. The similarities ended once stress and relapse were introduced.

The ketamine-like intervention, which rebuilt half of the lost synapses in a targeted way, stood out. After consolidation, the network with only 47.5% sparsity shrugged off the harshest stress (84.5% accuracy at σ = 2.5) and lost virtually no ground after a second pruning wave. This echoes clinical findings in which a single ketamine infusion triggers BDNF-dependent spine growth and can hold depression at bay long after the drug has cleared [4,5,14]. In the model, structural repair-not continuing drug action-accounted for the resilience, supporting the view that glutamatergic treatments create new "reserve" rather than simply damping symptoms.

The SSRI-like schedule told another story. Without adding new connections, it slowly fine-tuned the existing sparse network, pushing combined-stress accuracy from 31.8% to 83.5%. However, tolerance of severe noise stayed modest, and the network gave up 10.8% accuracy after the relapse simulation. That pattern fits the well-known therapeutic lag of monoaminergic drugs, whose benefits rely on gradual receptor and signalling changes rather than rapid synaptogenesis [15]. Network analyses of real trials likewise show that SSRIs first lift mood and anxiety; cognitive gains follow indirectly and are less robust [16,9]. The model, therefore, captures why these agents work reliably in milder illness but often fall short when plasticity is deeply impaired.

The neurosteroid-like condition added no new wiring yet instantly stabilised activity through stronger tonic inhibition. While the dampening was active, the network matched ketamine on the main stress test (97.5%) but collapsed once modulation stopped, confirming a strong state-dependence. Extreme internal noise was also harder to handle when inhibition was high (42.5%). Brexanolone and zuranolone exhibit an analogous trade-off in clinical settings-rapid relief that diminishes upon cessation of dosing [6]. The model indicates that these drugs merely prolong duration rather than restore reserves, which is advantageous in acute situations like postpartum depression.

**Figure 3:**
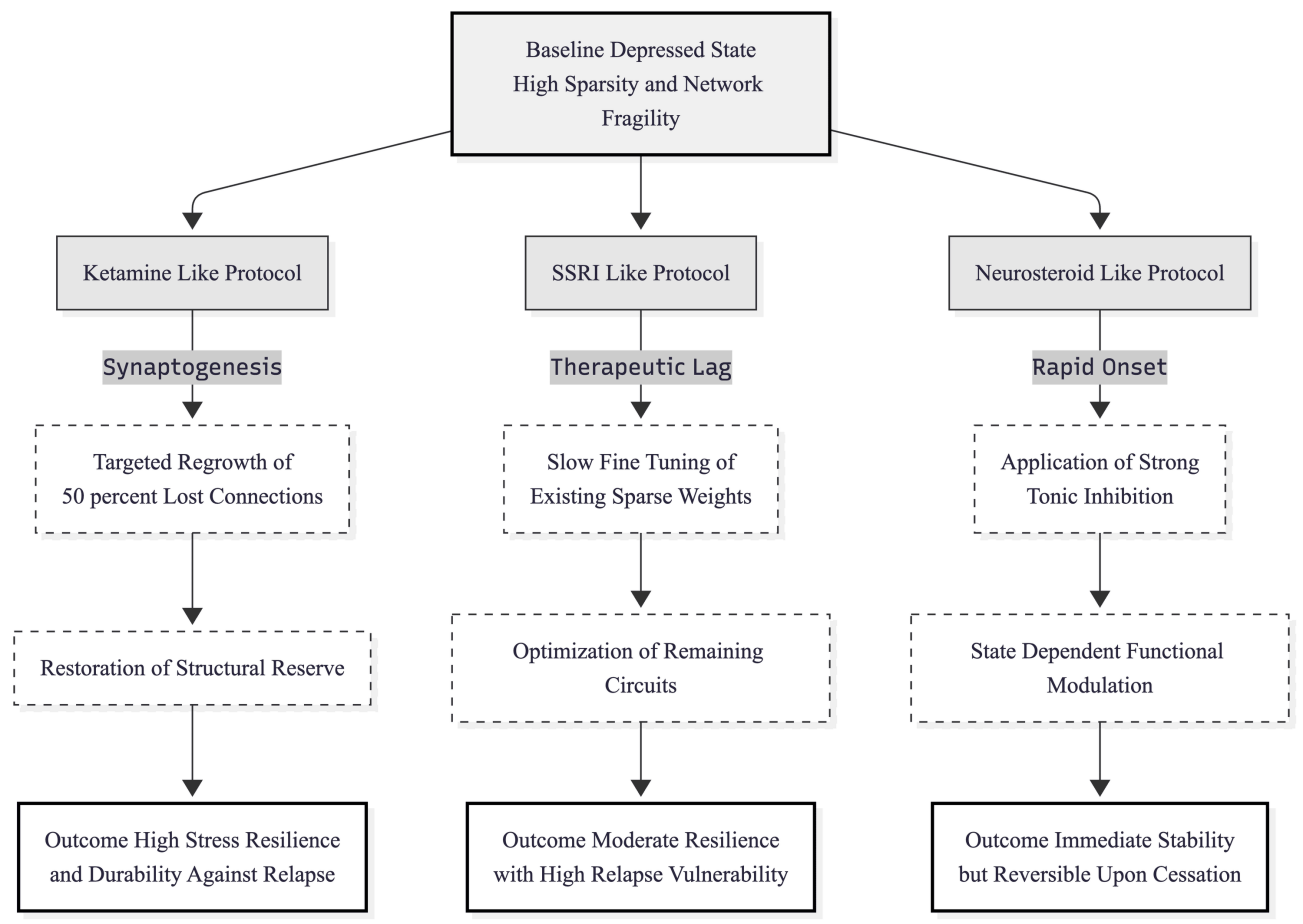
Comparative mechanisms of action and therapeutic outcomes in the modelled network The flow diagram illustrates how the three simulated protocols diverge from the baseline depressive state. The Ketamine-like pathway (left) relies on structural repair via synaptic regrowth, creating a "reserve" that confers long-term durability. The SSRI-like pathway (center) operates via the slow optimization of existing, sparse connectivity, resulting in functional recovery that remains vulnerable to severe stress and relapse. The Neurosteroid-like pathway (right) utilizes functional modulation via tonic inhibition, providing rapid but transient stability that is dependent on the active presence of the intervention. Image credits: Ngo Cheung (created using Mermaid)

Taken together, the findings reinforce a plasticity-based view of major depression [9]. Ketamine restores structural capacity, neurosteroids lend immediate but reversible stability, and SSRIs optimise what connectivity remains (Figure 3). Recognising these distinct routes can guide personalised care: patients showing deep structural loss may need synaptogenic agents first, whereas those with heightened excitability might respond to GABAergic modulation, and individuals with milder circuit deficits may still do well on monoaminergic therapy alone.

Novelty and translational impact

By placing glutamatergic, monoaminergic, and GABAergic strategies into the same pruning-based framework, the present study moves beyond earlier single-mechanism simulations. Starting each model from an identical, severely over-pruned "depressed" network allowed a clean comparison of three therapeutic routes: ketamine-like structural rebuilding, SSRI-like functional fine-tuning, and neurosteroid-like activity damping.

From those direct contrasts, several long-standing clinical puzzles begin to make mechanistic sense. Rapid, durable recovery after ketamine emerged because targeted synaptogenesis restored reserve and raised the failure threshold, mirroring sustained clinical benefit in otherwise refractory patients. The SSRI analogue acted more slowly and never fully matched ketamine under heavy stress, reflecting the gradual receptor and signalling adaptations seen in practice. The neurosteroid condition delivered the quickest gain but remained state-dependent, beneficial only while tonic inhibition was applied, capturing both the speed and relapse risk of brexanolone or zuranolone.

**Figure 4:**
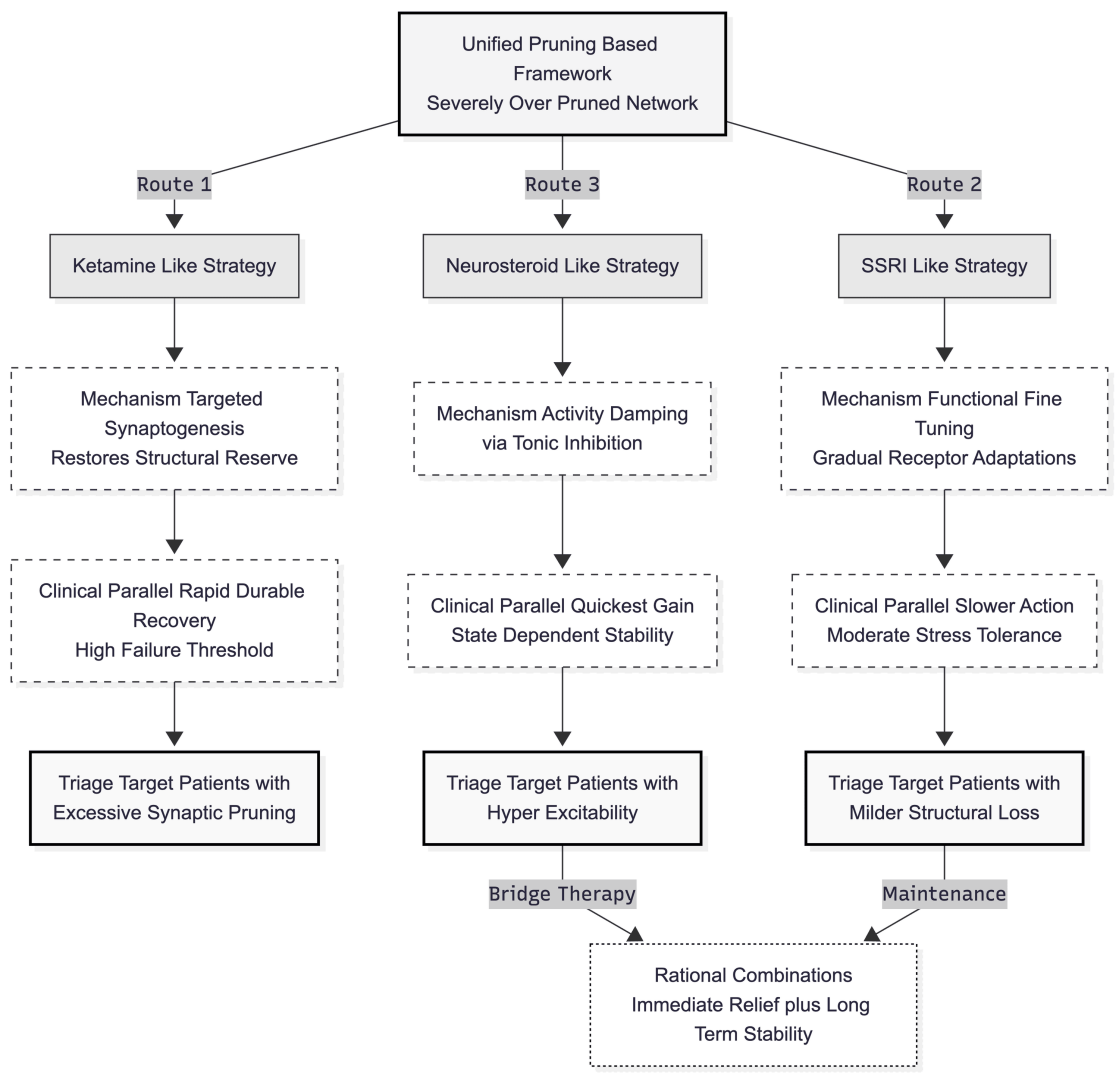
Translational implications and triage principles The diagram maps the three modelled therapeutic routes from their mechanistic origins to clinical applications. By distinguishing between structural rebuilding (Ketamine-like), functional fine-tuning (SSRI-like), and activity damping (Neurosteroid-like), the framework supports a precision medicine approach. Patients are triaged based on underlying neural deficits—structural loss versus hyper-excitability—facilitating rational combination therapies, such as using neurosteroids as a bridge to monoaminergic maintenance. Image credits: Ngo Cheung (created using Mermaid)

Such distinctions strengthen the neuroplasticity view of depression, shifting emphasis from a single transmitter deficit to the quality and quantity of synaptic connections [9]. They also suggest concrete triage principles (Figure 4). Individuals showing imaging or genomic evidence of excessive pruning might be channelled toward synaptogenic drugs; those with pronounced hyper-excitability could receive GABA-enhancing agents as a bridge; and patients with milder structural loss may still fare well with monoaminergic therapy. Finally, the framework hints at rational combinations, using neurosteroids for immediate relief while an SSRI consolidates longer-term stability, thereby addressing the stubborn non-response rates documented in sequential treatment trials.

Computational models such as the present one serve as vital bridges to precision psychiatry. By simulating mechanism-specific interventions in a controlled environment that would be ethically and practically infeasible in human trials, they generate testable hypotheses for biomarker-driven patient stratification (e.g., synaptic density via neuroimaging or excitability markers) and support rational, personalized antidepressant selection and combination strategies.

Supplementary insights from iso-dose comparisons

One concern with the primary comparisons was that the treatments differed not only in mechanism but also in the overall magnitude of network alteration-ketamine induced substantial synaptic turnover through regrowth, SSRIs relied on gradual weight shifts over many epochs, and neurosteroids primarily modified activation dynamics with minimal parameter change. To rule out confounding by unequal "therapeutic effort," we conducted parameter sweeps for each protocol and quantified change via the L1 norm of weight differences (normalized per parameter) as a cross-mechanism dose proxy. Configurations were then matched at overlapping dose levels (ranging from ~0.000855 to 0.007719), allowing direct evaluation of acute recovery, stress resilience, and relapse vulnerability under comparable network modification. This equalization largely preserved the core patterns: rapid gains were possible without structural change, but deeper robustness required more extensive alteration, particularly of the synaptogenic variety.

Across all matched dose levels, the ketamine-like protocol consistently excelled in preventing relapse, routinely showing the smallest (or even negative) performance drops after the second pruning challenge. At lower doses, where immediate combined-stress recovery was similar across modalities (~97-98%), ketamine still limited relapse to near zero (0.2% drop) while SSRI-like refinement lost up to 10.5% and neurosteroid modulation 4.5%. Even at higher doses, ketamine configurations occasionally gained accuracy post-pruning (−0.8% drop), underscoring the protective role of restored connectivity. These dose-controlled results reinforce that ketamine's advantage stems from genuine reserve building rather than sheer parameter turnover, aligning with observations that glutamatergic agents often sustain remission longer in recurrent or treatment-resistant cases.

Strengths, practical implications, and future research directions

The strengths of this study include the use of an identical severely pruned baseline for all three treatments, full public code availability, and direct head-to-head comparison of mechanistically distinct interventions, features rarely combined in prior computational work. Practically, the model provides a clear rationale for mechanism-based treatment selection and rational polypharmacy (e.g., short-term neurosteroid bridge + SSRI maintenance). Future directions include extending the framework to recurrent networks, incorporating individual variability via multiple seeds or patient-derived parameters, and validating predictions against neuroimaging or clinical trial data.

Limitations

The model remains an abstraction. A feed-forward network classifying four Gaussian clusters cannot capture the recurrent loops, neuromodulator gradients, or heterogeneous cell types present in corticolimbic circuits. Pruning and regrowth were implemented with magnitude thresholds and gradient ranking, omitting microglial, complement, and astrocytic processes that sculpt synapses in vivo. Likewise, tonic inhibition was represented by a simple global gain change; real neurosteroid action is layer-, receptor-, and state-dependent. Internal Gaussian noise served as a stand-in for stress hormones and inflammatory cascades but lacks their time courses. Finally, all simulations used the same seed, so interpersonal variability, central to clinical heterogeneity, was not addressed.

## Conclusions

Despite these simplifications, the work illustrates how fragile, over-pruned networks can be rescued by rebuilding, by patient fine-tuning, or by temporary damping, and why only the first route yields deep, relapse-proof resilience. Embedding diverse drug actions into a single developmental vulnerability model. therefore. offers a practical bridge between computational insights and bedside decisions. Future efforts that add recurrence, individual variability, and multi-stage regimens could sharpen those predictions and speed the arrival of faster, longer-lasting antidepressant care.
